# On Diseases of the Teeth

**Published:** 1829-10-01

**Authors:** 


### ON DISEASES OF THE TEETH. By *Thomas Bell*, F.R.S. &c.

[Concluded from No. 22, p. 336.]

IN the pursuance of our analysis of Mr. Bell's very excellent work, the next subject to which we come is—

### LOSS OF THE ENAMEL AND WEARING DOWN OF THE TEETH.

Mr. Hunter appears to have been the first to notice this "decay of the teeth by denudation," which consists of a gradual loss of substance, generally without discoloration, or any appearance of disease. It commences on the surface of the enamel, attacking first the central incisores, sometimes removing the anterior surface of the enamel generally or partially—in other cases making a perfect and regular horizontal groove, which gradually increases, till it has extended to the bone. From the central it passes on to the lateral incisores, and thence, in succession, to the cuspidati, the bicuspides, and, in some cases, even to the molares. The groove is perfectly straight and continuous through its whole extent, and is often as regular and smooth as if it had been formed by a round file and afterwards polished. When the bone of the tooth is exposed by this process, it becomes slightly discoloured, but remains long sound and hard, seldom exhibiting any appearance of gangrene. The cause of this affection has hitherto remained unknown. Mr. Bell thinks the most probable solution of the question would be the attributing it to friction, for he has seen many instances where it has appeared to be produced by the use of rough or acidulated tooth-powders, but in other cases no such cause existed.

"As I have here alluded to the mode which nature adopts to prevent the cavities of the teeth from being laid open by the removal of the bone, I will make a few observations on the wearing down of the teeth by mastication, to remedy which a similar process is set up. It often happens, either from the particular position in which the teeth meet each other, from a deficiency in the structure of the enamel, or from some peculiarity in the food, that the surfaces of the teeth are gradually worn away, until not only the bony structure is left unprotected by enamel, but at length the cavity itself would be exposed, but for a deposition of bone which is formed within it, in proportion to the external abrasion of its substance. The new bone is darker and more transparent than the original substance of the tooth, but nearly of the same degree of hardness. It is first deposited in that part of the cavity towards the worn surface, and becomes gradually more and more filled as the tooth becomes abraded. This process, as Mr. Hunter well observes, proves to demonstration that the opening at the extremity of the root does not close from age, except with the actual obliteration of the cavity by a fresh deposition of bone: for it is, undoubtedly, from the vessels of the internal membrane, supplied through that foramen, that this new bony matter is formed." 190.

II. *Salivary Calculus.* The deposition of a calculous deposit on the teeth is almost universal. When first deposited it is soft, friable, and readily crumbles under the finger; but, by a kind of slow crystallization, acquires almost a rocky hardness. It is most probably a deposition from the saliva.

"With the exception of gangrene, there is no kind of injury to which the teeth are exposed, so commonly and so extensively destructive as this concretion. As it is, generally, first of all deposited at the necks of the teeth, and especially underneath the free edge of the gum, its first effect is to excite more or less irritation in that structure, producing increased redness and sensibility, with sponginess and the separation of its edge from the necks of the teeth. As the accumulation increases, its effects keep pace with it; the gum becomes exceedingly painful, so as to render the ordinary operation of brushing the teeth almost impracticable, and thus, by inducing a neglect of the common means of preventing its accumulation, it becomes the unavoidable cause of its continued increase. Absorption of the gum and alveolar process is the next consequence, which gradually goes on until the teeth, losing their support, become loosened and at length fall out. A temporary and fallacious support is sometimes produced by a large quantity of tartar, which forms one continuous mass around the loosened teeth, and instances have occurred in which several teeth thus cemented together have come away without being separated from each other." 197.

In very irritable and unhealthy constitutions, a bad ulceration of the gums is thus induced, and sometimes extends to the cheek or tongue, rendering it necessary to remove the irritating substance, and to administer alterative remedies, combined with, or followed by, tonics, together with powerful astringent lotions to the parts.

"As this substance is formed from the saliva, whatever cause produces an unhealthy action in the glands secreting that fluid, will be found invariably to occasion a greater deposition of tartar. Fever—indigestion, or any irregular state of the stomach—drinking, smoking, and similar causes of irritation in the salivary glands, whether local or constitutional, immediate or sympathetic, will produce it. The action of mercury is one of the most speedy and unfailing causes of its accumulation, as it not only induces an increased and unhealthy action in the salivary glands, but also, by rendering the gums highly susceptible, prevents the necessary precautions from being used, by which the teeth might be kept clean.

"The earthy matter of salivary calculus combines with a considerable quantity of the mucus of the mouth during its deposition; and, as the same causes which occasion its formation, will tend equally to produce an increased and unhealthy secretion of mucus, which as it is thus rendered viscid, is with more difficulty removed, it often happens that this matter, already offensive from its diseased condition, becomes excessively fetid from putrid decomposition. Hence arises that peculiarly disgusting fætor which almost invariably accompanies the rapid deposition of tartar. If however the action which occasioned it be ckecked, this offensive smell gradually subsides as the calculous deposit becomes consolidated." 198.

In the way of prevention, a due attention to the state of the stomach will do much, but the accumulation of tartar can only be avoided by constant cleanliness, and by carefully removing it as soon as it is deposited, and before it assumes a consolidated state.

"The constant use of a tooth-brush will, in many cases, be sufficient to keep the teeth free from tartar. The brush should not be very hard, as it will not only be more difficult to clean the interstices between the teeth, the part in which the tartar is most likely to be deposited, but, by its friction, will occasion the gradual absorption of the gum, and the exposure of the necks of the teeth. The hair of the brush should be firm and elastic, and not too closely set. The teeth should be thoroughly brushed in every part at least night and morning, and the mouth always rinsed after each meal. In those constitutions in which there is a particular tendency to form tartar, it will be necessary to have recourse to some simple tooth-powder, such as prepared chalk, or any other substance equally simple and soft; it may, in some cases, be desirable to combine with it a small proportion of the bone of the cuttle-fish very finely powdered, and, if the gums are spongy and lax in their texture, a little alum, powdered myrrh, or bark may be added with advantage. Many of the tooth-powders which are offered for sale, with the promise of rendering the teeth beautifully white, perform, for a time, all that is promised, at the expense of permanent and irremediable injury to the teeth; for they often contain a quantity of tartaric or other acid, which effects a gradual decomposition of the enamel. The use of acids to the teeth cannot be too strongly deprecated. Even where it is necessary to administer acid medicine, it is of considerable consequence that it should be taken through a glass tube, to prevent it from acting upon the enamel of the teeth. For want of this simple precaution, the teeth are very often irremediably injured by the use of this class of remedies.

"The tartar is to be removed by means of instruments adapted for the purpose, and commonly known by the name of scaling instruments. They are of several forms, accommodated to the different situations from which the tartar is to be removed, and should be highly tempered, and the edges kept sharp and hard. It is of consequence that every particle of it should be taken away, not only from the external and internal surface of the teeth, but also between them; for if it be suffered to remain in any part, it forms a nucleus, around which a further accumulation will be immediately deposited. When the tartar exists in considerable quantities, and especially if the teeth are at all loosened, it is proper to remove it at different times, with an interval of some days, that the teeth may recover from the effects of the first operation before the second is performed, and in order that they may receive as much benefit as possible from this plan, the tartar which is formed around the necks of the teeth, and which has been the cause of the loss of the gum, and the consequent loosening of the teeth, should be first removed, which will allow of the gum being partially restored, and the teeth rendered, in some measure, firmer, and capable of bearing, without injury, the subsequent operations. In the mean time, this object will be much assisted by the frequent use of some astringent lotion, according to either of the following formulæ:

℞. "Aluminis, ʒiss.

Tinct. Myrrhæ, fʒiii.

Mist. Camphoræ, f℥vss. Misce.

℞. "Vini Rubri Lusitan.
Mist. Camphoræ, ā f℥ii. Misce.

℞. "Infusi Rosæ, f℥ii.
Decoct. Cinchonæ, f℥iv. Misce." 201.

After the removal of the tartar, if the gums should remain swollen and spongy, they should be occasionally scarified, the foregoing lotions being continued for a time. When much heat and pain are felt in the gums, a solution of nitre in barley water is a useful fluid for washing the mouth. The application of strong mineral acids for the removal of the tartar is to be deprecated as excessively injurious, as it dissolves the enamel and the earthy part of the bone whenever it comes in contact with them.

III. *Diseases of the Internal Membrane.* The frequent occurrence of inflammation in a structure so delicate and exquisitely sensible as the internal pulp or membrane of the tooth, need not be wondered at; and the results of such inflammation, surrounded by hard unyielding substance, must be severe.

"The suffering occasioned by inflammation in a sound and previously healthy tooth is, in fact, of a very different character from that which is produced by the exposure of the membrane by fracture or by decay. In the former case, it is of a deep heavy kind, and wholly unaccompanied by those acute darting paroxysms which attend the exposure of the pulp; though, with that exception, it is scarcely more endurable than severe and continued tooth-ache, properly so called. Whenever a tooth becomes inflamed, from whatever cause, and the pain which I have now described occurs, the gum should be repeatedly bled by leeches; and, if they are early applied, and the bleeding be freely encouraged, the inflammation will generally be soon arrested, and no unpleasant consequences ensue. But it occasionally happens that the inflammation goes on to such an extent as to produce either alveolar abscess, or even suppuration of the pulp itself, and the consequent confinement of an increasing secretion of pus within the dental cavity. This constitutes the disease which Mr. Fox considered as resembling *spina ven-*

*tosa*, though certainly it requires some stretch of the imagination to see the analogy between these two affections.

"The matter, by its pressure, produces absorption in the parietes of the cavity, and at length finds an outlet by this means at the extremity of the root, the foramen of which is very greatly enlarged. The pulp has by this time become partially absorbed, and the remaining portion having mortified, the tooth also loses its vitality, and gradually assumes a darkish hue. The pus having now escaped into the extremity of the alveolar cavity, at which part the thickening of the periosteum and effusion of lymph had formed a solid organized mass, this becomes absorbed in the centre, and forms the sac of the abscess, which now differs in no respect from common alveolar abscess, excepting in its communication with the dental cavity. Absorption of the alveolar process and gum gradually gives an outlet to the enclosed pus, and a discharge continues afterwards to take place, which, from the mortification of the pulp, is so extremely fetid, as to form a tolerable diagnostic character between this disease and common alveolar abscess occurring from inflammation of the alveolar periosteum, in which the pus is usually nearly inodorous, excepting when it has been long confined, or when the bone has become carious. On extracting the tooth, the only remedy when pus has once been formed, the tooth will generally be found absorbed in patches, not only within the cavity, but on the external surface of the root also, as in all cases of total necrosis of a tooth from any other cause. Should the tooth be retained, the opening made by the pus will remain fistulous, and the matter continue constantly to be discharged. The termination of such cases is, in fact, precisely similar to that of total necrosis, as already described." 205.

IV. *Diseases of the Gums and Alveolar Processes.* The greater number of these are produced by the irritation arising from diseased or dead teeth —and it is rare to see a strictly idiopathic affection of these parts. But the sympathetic diseases are much modified by idiosyncrasy; for what in one person would only produce simple abscess, will, in another, lead to ulceration of a decidedly cancerous type, or fungus, &c. Constitutional disorder will sometimes also affect the gums and alveolar processes, independent of diseased teeth.

"A cachectic condition of the system, the abuse of mercury, and similar causes, will frequently bring it into action; but perhaps no persons are more liable to its occurrence than those in whom the constitution is worn out by debauchery and intemperance, where the system has become irritable in proportion to its debility, and where the tone of the stomach has yielded to the effects of a long-continued course of incessant excitement; and the disease assumes a more or less formidable character, according to the extent to which the constitution has suffered." 208.

But as this subject belongs to general pathology rather than to dental surgery, Mr. Bell proceeds to points more closely connected with the object of the publication. It is curious, he observes, that absorption of the gum never precedes that of the alveolar process, at least so as to occasion denudation of the edge of the bone. On the contrary, the bone is always removed first. This alveolar absorption is, of course, a natural process in old age, and is then merely an indication of general decay. It sometimes, however, takes place even before the middle period of life, indicating a premature decline of the powers of life.

"This disease is very frequently first excited, and afterwards kept up, by continued indigestion, or some affection of the stomach, constantly acting upon the system. In other instances the cause may have existed at a remote period; and the action, which was then set up in the gums, having become chronic, the local disease continues long after the cause which had produced it ceases to exist. Thus an attack of fever, frequent pregnancies, and any other violent or repeated commotion in the system; but more than all, the action of mercury carried to excess, will very often be found to have originally produced the disease, which survives its cause, in many instances, for a great length of time. I have seen numerous cases in which the action of mercury had apparently ceased soon after the cure of the complaint for which it was administered; yet, after the lapse of two years or more, the gums and alveolar processes have reassumed a morbid action, and this has gone on to such an extent as to occasion the loss of the greater number of the teeth.

"The local symptoms of the disease in question resemble, in a great measure, those of all other diseases of the gums arising from irritation. The gums are spongy, turgid, and vascular, bleeding very readily on being pressed, and generally very painful to the touch. In some cases, the edges only are thickened, the enlargement being distinctly circumscribed; in others, the swelling extends to the whole substance of the gum. The colour is very much deepened, and of a purplish hue, with a slight degree of transparency, which gives a very peculiar appearance, not easily described. If this state of the parts be suffered to remain unattended to, the symptoms gradually become more aggravated. The irritation extends to the interior of the alveoli, occasioning a thickened state of the periosteum, with a slight degree of loosening of the teeth, the pressure of which produces more or less pain. The alveolar or dental periosteum at length secretes a purulent matter, which is discharged at the necks of the teeth. The alveolar processes continue to be gradually absorbed; but so slowly, that, in some cases, several years may elapse without producing much alteration. At length, however, their absorption extends so far as no longer to allow of their retaining the teeth, which, one after another, spontaneously fall out. In general, the gum very speedily assumes a healthy appearance after the loss of these loose teeth; but I have seen a few cases, in which even the part from which the teeth have been removed, continues in an unhealthy state, which defies every local as well as constitutional remedy, which has been resorted to for its cure." 212.

The treatment of these affections is simple; but it should be decisive and prompt. The first thing is to lance the gums *freely*. It is not upon the gradual abstraction of a certain quantity of blood that the advantage of this operation depends, but upon the sudden and perfect unloading of the vessels of the spongy gum, and the contraction of those vessels immediately consequent upon it.

"The best mode of fulfilling this object, is by passing a common lancet into the gum, carrying it between the teeth as low as the transverse alveolar processes will permit, and thus making a complete perpendicular section of each portion of gum: the lancet is then also to be drawn across the gum horizontally; and it will be found, if the lancet be carried sufficiently deep, that an immediate collapse of the vessels will take place; and this is to be rendered permanent by the frequent use of some strong astringent lotion. The following formula may be used for this purpose:

"℞. Aluminis Sulph. ʒij.

Decoct. Cinchonæ

Infusi Rosæ ā ℥ij. Misce. Fiat lotio.

"The section of the gums should be repeated from time to time at intervals of a week, until the sponginess and inflammation have entirely subsided; and the necessity for a frequent and continued use of this plan will, of course, depend upon the degree and obstinacy of the disease." 215.

In all cases it is highly necessary to pay great attention to the stomach.

Mild alteratives should be given till the fur of the tongue disappears, and the bowels are restored to healthy function, after which tonics will be very beneficial.

V. *Alveolar Abscess.* The term gum-boil conveys a most erroneous meaning. The gum is only secondarily affected, the cause being invariably within the alveolus. It is produced in various ways—sometimes, though rarely, by inflammation in the periosteum of a sound tooth, from cold or other local cause. The irritation of tooth-ache is a common cause—but the most frequent cause is the existence of a dead tooth or root acting as an extraneous body in the socket.

" The first effect produced by the irritation of a diseased tooth, or a dead root, is a thickening of the periosteum of the alveolar cavity, which raises the tooth in the socket, and renders it a little loose, and susceptible of considerable pain on pressure: an effusion of coagulable lymph then takes place around the extremity of the fang, which becomes condensed into a sac, in the centre of which pus is formed. The sac will be found closely embracing the root just above its extremity, which is, as it were, bathed in the pus." 216.

" The irritation increases, and extends to the gum and neighbouring parts, which become affected with severe throbbing pain; redness, and thickening of the gum, and at length a greater or less degree of tumefaction of the face occurs; and the pus, having occasioned by its pressure absorption of the investing parts, forms for itself an outlet, in some cases externally, either in the cheek, or opposite the base of the lower jaw, but more frequently within the mouth through the gum." 217.

The treatment is similar to abscess in other parts. Before the actual formation of pus, leeches should be freely applied—and aperients given, aided by abstemious diet. If matter forms, its exit should be promoted through the gum by fomentations in the mouth, and cold applications to the face.

As soon as the abscess has approached the surface, the pus should be evacuated. This treatment, however, is only palliative; for, as long as the roots remain in the socket, the opening, through which the matter has passed, will continue fistulous, and pus will ooze through it. The removal of these roots will prove the permanent cure of the disease.

We must pass over several instructive sections of the work before us, and only glance at a few of the subjects en passant.

VI. *Effects of Mercury on the Teeth and Gums.* The irritation produced in the gums is a pretty fair criterion of the constitutional effects of mercury. It is well known that, in some constitutions, a single dose of mercury will induce ptyalism.

" From the very general use of this medicine, and still more from the careless and profuse manner in which it is sometimes administered, it may be considered as one of the most common causes of the diseases, to which the teeth and their containing parts are liable. I have already alluded more than once to its influence, in producing a tendency to gangrene as well as total necrosis of the teeth—inflammation, and consequent suppuration in the alveolar periosteum alveolar abscess—sponginess, ulceration, and absorption of the gums, the deposition of salivary calculus, &c. But these, although the most frequent consequences of an excessive use of mercury, or of its more moderate administration under a peculiar idiosyncrasy of constitution, are not the only, or the most severe results. Caries and necrosis of large portions of the alveolar process, sometimes extending far into the body of the bone, are occasionally found to follow a profuse course of this medicine. In the diseases of infancy and childhood, mercury is too often administered by the mother or the nurse, with a degree of careless excess which ultimately, if not immediately, produces severe and irremediable injury." 245.

Sloughing of portions of the gum, accompanied by extensive ulcerations surrounding the dead part, is another consequence of excessive mercurial action, the constitutional irritation resulting from which, is sometimes very severe.

"In cases of excessive sponginess of the gum, whether accompanied with ulceration or not, in thickening of the alveolar periosteum, and other similar affections, no treatment will be found effectual, until the medicine itself is discontinued. The use of astringent lotions will be found serviceable, and the excessive and disgusting fætor may be corrected by the frequent use of a wash composed of the chloruret of lime or of soda, very much diluted. It is not, however, whilst the mercurial action is still kept up in the system, that the only remedy to be depended upon for the permanent relief of the gums, can, with propriety, be had recourse to; namely, the free and deep application of the lancet. But when the medicine has ceased to act, and the swelling and other symptoms have assumed a chronic form, the vessels of the gums should be unloaded by carrying the lancet freely through their substance, down to the alveolar processes; and the collapsed state of these parts may then be in a great measure preserved by the use of strong astringents. The bleeding should be repeated as soon as a return to the former turgid state is observed in the gum. If the lancet be used during the action of the medicine, the result will very often be the occurrence of unhealthy ulceration, which it will be very difficult to heal, as long as the mercury is persevered in." 247.

VII. *Disease of the Antrum Maxillare.* The mucous membrane lining this cavity is readily excited to inflammation by any cause acting on the pituitary membrane of the nose—but especially from irritation produced by the teeth.

"When inflammation of this cavity is produced by a tooth—whether from the exposure of its internal membrane, if the tooth be living; or, if dead, by the irritation which it excites as an extraneous body—it often assumes a more severe and important character, both in its present symptoms and its future results. The teeth which are most commonly found to occasion inflammation in the antrum, are, as might naturally be inferred from their situation, the first and second molares, and the second bicuspis; though it sometimes happens that the first bicuspis, or even the cuspidatus, and, still more rarely, the dens sapientiæ will produce this effect. When, from constitutional irritability, arising from derangement of the digestive organs, general debility, or any other cause, irritation is produced in the sockets of any of the teeth now mentioned, the usual symptoms which precede alveolar abscess, and which have already been described, are often followed by the occurrence of deep seated pain in the body of the bone, which, although generally of a dull, heavy, aching character, is frequently interrupted by acute lancinating paroxysms darting across the cheek towards the interior of the nostril, and backwards in the direction of the tubercle of the maxillary bone; this is accompanied with a greater or less degree of general tumefaction of the cheek, and with circumscribed patches of a florid colour upon the skin; and these parts, as well as the integuments and periosteum of the surrounding bone, are more or less painful on being pressed.

"The inflammation should be at once reduced by the free application of leeches to the cheek; the state of the stomach and bowels must be carefully attended to, and the tooth or root, which had produced the irritation, should be immediately extracted." 252.

If the case be neglected, the mucous secretion from the lining membrane becomes altered in its character, assuming a purulent form. The natural opening into the nose becomes obliterated—sometimes permanently so, by adhesive inflammation. The consequent retention of the mucous secretion ultimately produces caries of some part of the parietes.

"The term abscess, as applied to the disease in question, has given rise to very mistaken notions of its nature, and not less erroneous principles of treatment. A reference to the structure of the antrum would appear to be sufficient to point out the improbability, to say the least, of the occurrence of abscess in such a situation. That a mucous membrane covering, in a thin layer, the whole internal surface of such a cavity, should become the seat of all the consecutive steps of true abscess, is a statement bearing on the face of it an obvious absurdity. The disease is, in fact, nothing more than an altered secretion of the membrane. In its healthy state, this fluid is exactly similar to that which is formed by the Schneiderian membrane; it is equally fluid, inodorous, and transparent. The first effect of inflammation is to increase its quantity, and afterwards to render it thicker and more tenacious. It frequently becomes even glairy in its consistence; but, in this respect, it is liable to considerable variation. At length it assumes an opaque whitish or yellowish colour, and resembles in every respect true pus. Whether this change is confined to an alteration simply in the natural secretion of the part, or whether some of the mucous follicles may become the subjects of suppuration, and thus a mixture of mucus and of true pus be formed in the antrum, is, perhaps, in some measure doubtful. But that the former is most probably the true state of the case is, I think, supported by the gradual steps by which the secretion becomes changed, as described above. In proportion to the degree of inflammation which has existed—its duration—or the confinement of the purulent secretion within the cavity, it becomes more or less fetid; and, in cases where the nasal opening has been long closed, the fætor, when the matter is evacuated, is intolerable, even where no disease has taken place in the bone." 254.

It is usually a considerable time after the occurrence of inflammation before the secretion becomes puriform in a decided and unequivocal manner. Mean time the symptoms before described increase in intensity—the pain is more severe—the cheek becomes more tumid—the redness augments—and, if the nasal opening has not been obliterated, a discharge of purulent mucus takes place from the nostril—especially when the patient lies on the opposite side. In this state things may long remain, with little other variation than increase or decrease of the discharge, arising from variations of the health, or other adventitious circumstances. But, in scrofulous or very debilitated constitutions, caries or exfoliation of the bone will sometimes take place. When an early obliteration of the nasal opening obtains, and the matter is pent up, an enlargement of some part of the parietes of the antrum ensues, attended with much distress and deformity. The usual termination of such cases is caries and exfoliation of some part of the bony parietes, with fistulous opening on the cheek.

"*Treatment*. From the account which has been given of the nature of this disease, it will be obvious that the indications which are required to be fulfilled in its cure are threefold. The first is the evacuation of the contained fluid, in those cases where the nasal opening is closed; secondly, the correction of the diseased condition of the membrane; and, thirdly, the removal of such portions of bone as may have become carious.

"The opening of the antrum for the purpose of allowing the escape of the accumulated fluid, is generally a very simple operation. It consists in most cases of nothing more than the introduction of a trocar of a suitable description into the antrum, through the alveolar cavity of some one of the teeth, which are situated immediately beneath it. The trocar which is generally used for this purpose, is bent at a right angle at about an inch from the point; but I have always found this a very inconvenient and tedious instrument. I have, for several years, used a perfectly straight one, with greater facility and expedition; and the direction of the alveoli of the upper jaw is so favourable for its introduction, that I have never found the least difficulty in opening the mouth sufficiently wide to enable me to avoid any interference with the teeth of the lower jaw, or with the cheek. The point is three-sided, and should be very sharp." 260.

For the first few days warm water only should be injected, and leeches, if necessary, be applied—after which stimulating injections are to be thrown up, as a solution of sulphate of zinc, or tincture of myrrh.

VIII. *Neuralgia.* Our space being nearly filled up, we are obliged to pass over several sections, in order to devote a page or two to the painful affection now under consideration. The object of Mr. Bell, in this section, is to assist in forming some discrimination between two distinct forms of neuralgia, "which have hitherto been confounded with each other," and thus to lead to a more certain prognosis and a less empirical method of treatment.

"Having for some years past paid no inconsiderable attention to the different forms of the complaint, I have long since come to the conclusion that two distinct affections—distinct in their causes, and equally so in the effect which different remedies are found to exert upon them—have been confounded under these terms; the one constitutional in its cause, and curable by general remedies; the other local, which, though occasionally relieved to a certain degree by such treatment, can only be permanently cured by the removal of the local cause, which, unhappily, often lies too deep to be within the reach of any operation.

"It must be confessed that the symptoms of these two affections are so similar as to be readily confounded, and perhaps it would be difficult to lay down any one character which should infallibly distinguish the one from the other. There are, however, some general diagnostic symptoms which, after having seen many cases, will, I believe, enable the practitioner to decide with considerable certainty in any well-marked case.

"In those which have a constitutional origin, periodical returns of the paroxysms are, if my views be correct, an invariable symptom. This may vary to a great extent, not only in the degree, but also in the regularity which may characterize the exacerbation of pain. In some cases the pain is incessant, and almost always equally severe; and it is only by careful attention, that its periodical increase can be ascertained. In others, not the slightest painful sensation is perceived during the twenty-four hours, with the exception of a distinct and certain period, which never fails, and never varies, and during which perhaps the agony is intense.

"In the local disease, on the contrary, the pain is either continual, without any marked paroxysms of increased suffering; or the attacks are sudden, frequent, and interrupted by intervals of perfect ease, but recurring from time to time, and, in both cases, without any regular periods. This is the most obvious diagnostic character which has appeared to me to distinguish the two diseases. Further observations may perhaps enable us to add other and more certain marks of distinction; but even this will, as I believe, be found generally sufficient to indicate the nature of the disease." 312.

In respect to the constitutional neuralgia, it appears to our author that no cause acts more frequently in producing, or at least increasing the disease than violent mental agitation, or long-continued anxiety. Numerous cases have come under Mr. Bell's notice, where the origin of the disease could be traced to loss of friends, reverses of fortune, disappointed hopes, and similar causes of mental depression.

The recurrence of such circumstances during intervals of relief is almost sure to produce relapses of the complaint.

"There is one circumstance which is of the utmost consequence to attend to in this complaint, and which is an universal attendant upon its attacks, whether as a cause or a consequence. I mean a more or less deranged state of the digestive functions. I never saw a case of constitutional neuralgia without a white tongue; and I never knew one cured by any of the usual constitutional remedies, until the digestive organs were restored to a more healthy condition." 316.

The publications of Mr. Hutchinson induced a general adoption of carbonate of iron, as a remedy in neuralgia, but it was found often to fail—perhaps from inattention to the chylopoietic functions rather than to the inefficacy of the medicine.

"In many cases in which this remedy has failed, I fave found the use of the sulphate of quinine completely successful. This admirable medicine, possessing all the virtues of the cinchona without the disadvantage of its bulk, by which the stomach was so often injuriously loaded, is one of the most important acquisitions in the treatment of this disease. I could mention numerous examples in which it has been entirely successful, and many others which have been materially benefited by its use; but the following case, in which no other remedy was employed, will be sufficient:—

"Mrs. W., a lady about fifty years of age, had for four years been suffering the most excruciating torture from neuralgia, which attacked the branches of the suborbitary nerve. The paroxysms were periodical and perfectly regular. They occurred invariably at the hour when the family usually took tea, and it frequently happened that she could not remain at the table on account of the severity of the pain. It rarely occurred at any other period with any considerable violence, but occasionally in case of indisposition or of sudden mental excitement, it would come on at any other time; this, however, never interfered with its regular recurrence at the stated period which I have mentioned. The first attack of the disease had been produced by extreme mental agitation arising from the sudden death of a dear friend. When I first saw her, she had been under the care of her medical attendant, who had administered large quantities of the carbonate of iron without any advantage. The state of the stomach and bowels had not been sufficiently attended to. The tongue was considerably furred, the bowels habitually costive, and the appetite uncertain.

"As the attacks were so regularly periodical, and as my patient was naturally of a tranquil and cheerful disposition, I could not but hope for a favourable termination of the case. The first step which was taken was to regulate the state of the bowels, for which the following mild formula was found sufficient:—

℞. Pil. Hydrarg.

Extr. Hyosciami, ā. gr. ij.

Extr. Colocynth. Comp. gr. iv. M. fiat massa, in Pilulas duas dividenda, alternâ nocte sumend.

"After a few days I ordered the sulphate of quinine to be taken in doses of three grains, three times in the day, and as it was essential to keep the mind as tranquil as possible, and to prevent it from musing upon subjects of a depressing nature, a short tour, in company with part of her family, was resolved upon. The remedies above mentioned were continued with great regularity and with the most complete success. In the course of three months the complaint was entirely removed, and has not since returned." 318.

Our author, however, like most other practitioners, has met with cases which resisted the quinine. In some of these arsenic was effectual. It appears that Mr. Bell himself was a martyr to neuralgia during a long period of thirteen years, and was ultimately cured by arsenic. Mr. B. has recorded a sketch of his own case, and, as it is short, we shall here insert it.

"It was, to the best of my recollection, in the spring of the year 1812, that I was attacked when walking, with a most acute and severe pain in the left heel, about the spot of the insertion of the plantaris muscle. It was of a fluttering character, with sudden and repeated flashes of a more acute kind, which were but momentary in their duration. This first attack remained about a quarter of an hour, and then wholly disappeared. It recurred, however, at intervals, with more or less regularity, until at length it assumed a more perfect intermittent character, the paroxysms returning every evening; and this occurred with little variation during the whole period of its continuance. During the paroxysms the skin was somewhat redder than usual, and a little swollen; and the surface was excessively irritable, especially when lightly touched. At all other times, however, it was perfectly healthy, and did not at all interfere with the action of walking, nor did previous violent exercise appear to increase the severity of the pain. The duration of the attacks varied from a fortnight to three months; during which periods an evening seldom passed without several hours of absolute torture, and the intervals also varied in a similar manner; I have repeatedly passed two or three months without pain, and on one occasion it did not return for more than a year. It was invariably brought on by the occurrence of any source of mental agitation or depression; and continued a longer or shorter time, according to the degree or duration of such exciting cause. The local remedies which I had recourse to were leeches, perpetual blisters, electricity, belladonna plasters, and a multitude of others, all equally useless. The only thing which gave me even temporary relief, was plunging my heel into the coldest water that could be procured; this relieved the pain as long as the part continued cold, but as soon as it became warm again, the pain returned with the same violence as before. I had recourse, at different times, to various remedies directed to the constitution: cinchona in powder, carbonate of iron, sulphate of quinine, &c., were all freely and fully tried, and all without success; at length, at the commencement of a rather violent attack in 1826, I was induced at the earnest instigation of my friend, Mr. Leese, to try the arsenical solution. The agony which I suffered, led me to take larger doses than my kind friend advised, and than, perhaps, would generally be advisable, at least at the commencement. I took eight drops three times a day, for about a week, at which time the stomach became excessively irritable, the whole nervous system was in a state of morbid excitement—I was obliged at once to discontinue the medicine, and to go into the country; but the disease was cured, and to the present hour I have had no return." 320.

The irritating effects of arsenic on the stomach and nervous system would render it prudent to try other means first.

On local neuralgia, or that caused by local injuries, pressure, or other sources of irritation, we need not dwell. The sagacity of the practitioner will be required to detect the nature and seat of the local cause, and no general rule can be laid down. We must now take our leave of Mr. Bell, whose work, we have no doubt, will become a class-book on the important subject of dental surgery.

